# EMG versus US: a randomized clinical trial comparing the efficacy in guiding botulinum toxin treatment in cervical dystonia

**DOI:** 10.1055/s-0045-1809542

**Published:** 2025-06-20

**Authors:** Mayara Thouin Graciani, Flávio Henrique de Rezende Costa, Ana Lucia Zuma de Rosso, Gil Fernando Salles

**Affiliations:** 1 Universidade Federal do Rio de Janeiro, Faculdade de Medicina, Departamento de Neurologia, Rio de Janeiro RJ, Brazil.; 2 Universidade Federal do Rio de Janeiro, Faculdade de Medicina, Departamento de Medicina Interna, Rio de Janeiro RJ, Brazil.

**Keywords:** Botulinum Toxins, Torticollis, Electromyography, Ultrasonography

## Abstract

**Background:**

Botulinum toxin type A (BoNT-A) is considered the first-line therapy for cervical dystonia.

**Objective:**

To compare, in a randomized trial, the efficacy of treatment with BoNT-A guided by ultrasound (US) and electromyography (EMG) in patients with idiopathic cervical dystonia.

**Methods:**

A total of 40 patients (20 in each group; mean age: 54 years; 45% of female subjects; mean disease duration: 10.7 years) were randomized to either US- or EMG-guided BoNT-A treatment. The efficacy of BoNT-A was assessed through changes in the scores on the Toronto Western Spasmodic Torticollis Rating Scale (TWSTRS) before and 4 to 6 weeks after the treatment. The differences in the absolute and relative changes in the total TWSTRS scores and in its components (severity, incapacity, and pain) between the groups were evaluated.

**Results:**

The US and EMG groups were well balanced in relation to baseline and demographic characteristics. After the BoNT-A treatment, there was a mean reduction in the TWSTRS score of 8 points (relative reduction of 23%) equally between the US and EMG groups (mean difference in absolute decrease of 0.1 point; *p* = 0.97; and mean difference in relative decrease of 2%; *p* = 0.89). There were no differences in the declines in the scores on the TWSTRS components, nor when the improvements in the TWSTRS scores were dichotomized as more significant or lower reductions (all *p* -values > 0.3).

**Conclusion:**

The present randomized trial did not demonstrate any difference in improvements between BoNT-A treatment guided by US or EMG in patients with idiopathic cervical dystonia.

**Clinical Trial Registration:**

ReBEC Identifier: RBR-33dd4p4.

## INTRODUCTION

Cervical dystonia (CD) is the most common form of focal dystonia, and it is a highly-disabling condition characterized by involuntary, twisting, sometimes tremulous head and neck movements, usually painful. 1 2

Currently, botulinum toxin type A (BoNT-A) is considered the first-line therapy for this condition; 3 it blocks acetylcholine exocytosis at the neuromuscular junction with the onset of effect after 7 to 10 days of the injection, a maximum peak in 2 to 6 weeks, and it begins to wear-off in 2 to 3 months after the injection. 4 The success of BoNT-A injection for the treatment of CD depends on the precise identification of the dystonic muscles and the quantification of their dystonic involvement, 5 6 as well as the involvement of deep cervical muscles. 7

Injections of BoNT-A guided either by ultrasound (US) or electromyography (EMG) are superior to injections performed using anatomical guidance, 8 9 10 11 although in the hands of experienced injectors, the results might be similar. 12 While there is general agreement that US guidance provides greater anatomical accuracy for BoNT-A injections, additional data are needed to assess its superiority over other guidance techniques. 12 13 14 To date, no head-to-head clinical trials have compared the efficacy of the two methods (US and EMG); thus it is not known whether there is a method superiority concerning the efficacy of BoNT-A injections in CD.

Ultrasound (US) offers several advantages over other guidance techniques, including the ability to track the needle's path, thereby avoiding nearby structures such as bones, blood vessels, and nerves, and the ability to visually check whether the needle is inserted into the target muscle, as well as to identify muscle fibrosis or contractures. It is also an economically-advantageous technique, as it does not use expensive monopolar electrodes. 15 16 Electromyographyhas commonly been used to detect dystonic muscles or to direct the appropriate muscles for botulinum toxin (BoNT) injections in patients with CD. 4 5 However, it is not possible to verify whether the needle is located in the target muscle without imaging guidance, such as US. 17 18 Additionally, anatomical variations, obesity, abnormal neck and head posture, as well as atrophy of the neck muscles, can also increase the risk of incorrect needle placement using EMG or palpation guidance. 12 17 The neck muscles are small and thin, and some have a complex orientation compared with the muscles of the extremities. Furthermore, several vital neurovascular structures pass through the neck and thorax in close proximity to these muscles. Therefore, imaging guidance is highly recommended. 19 Electromyography can be used in the preinjection stage, that is, in the assessment of dystonic muscles and in the planning of treatment with BoNT; it enables the examiner to distinguish those muscles that are contracting involuntarily from those that are not, in which injection should be avoided. 4 The precise location of the muscles minimizes the number of injections and the amount of BoNT, thus reducing the risk of antibody development and decreasing the effect on non-injected muscles. 20 21

The severity and features of CD are typically assessed using the Toronto Western Spasmodic Torticollis Rating Scale (TWSTRS), 22 23 24 which is composed of three subscales that measure symptom severity, disability, and pain. The TWSTRS was developed specifically for patients with CD. 23 24

The main goal of the present study is to compare, in a randomized trial, the efficacy of two well-recognized BoNT-A injection-guided techniques, US and EMG, in subjects with idiopathic CD.

## METHODS

### Study overview

**Figure 1:**
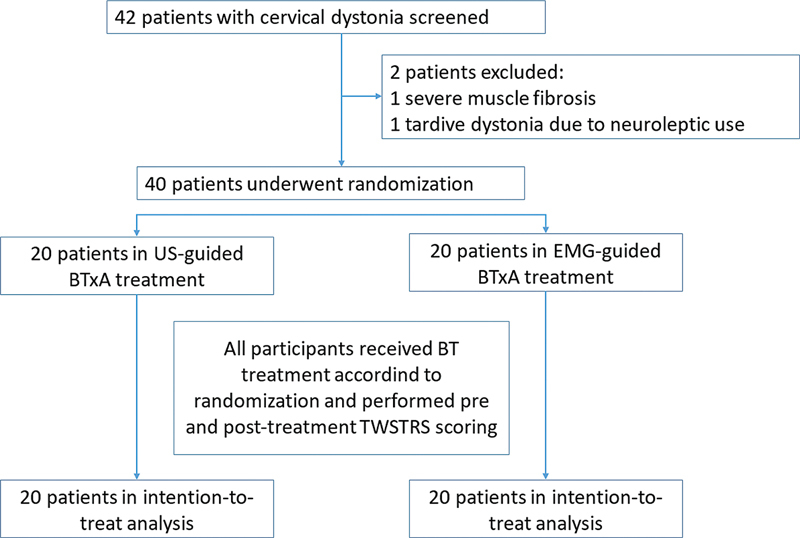
Abbreviations: BoNT-A, botulinum toxin type A; EMG, electromyography; TWSTRS, Toronto Western Spasmodic Torticollis Rating Scale; US, ultrasound. Study flowchart.

The current is a single-center, randomized, open-label, parallel-group clinical trial aiming to detect superiority through a blinded assessment of outcomes, conducted at a tertiary-care University Hospital to compare the efficacy (thorugh the TWSTRS) of US and EMG in guiding BoNT-A injections in patients with idiopathic CD. All treatments were administered by a neurologist who did not participate in the investigation procedures. The physician raters were blinded to treatment assignment. After obtaining baseline measurements, patients meeting the study criteria were randomly divided into two groups. Group A received BoNT-A injections guided by US, whereas group B received BoNT-A injections guided by EMG. The patients enrolled were randomized to either group through sorting sealed opaque envelopes in blocks of 4 participants stratified by sex and age (> 50 and ≤ 50 years). The study flowchart is shown in Figure 1 . The patients' follow-up visits were conducted 4 to 6 weeks after the injection. The patients were free to discontinue the trial at any time during the study. Those who were on anticholinergics, benzodiazepines, muscle relaxants, or analgesic medications were requested to remain on the baseline medication doses for the duration of the study.

The inclusion criteria were patients with idiopathic CD, of both sexes, aged between 18 and 80 years, and with a maximum disease duration of 20 years. The exclusion criteria were the presence of other movement disorders, fixed dystonia due to injection-related fibrosis, severe muscle atrophy, any cognitive impairment, any medical condition or use of any agent that might put them at increased risk if exposed to BoNT-A (such as neuromuscular disorders or agents that might interfere with neuromuscular function), and conditions that increase the risk of adverse effects, such as clotting disorders or previous adverse reactions to BoNT-A injections.

The BoNT-A injections were performed using abobotulinumtoxin A – Dysport (Ipsen Limited), 500 units/vial, diluted in 2.5 mL of 0.9% saline solution, at a concentration of 200 units/mL.

All patients enrolled provided written and signed informed consent. The local Ethics Committee had previously approved the study protocol (under CAAE 28311219.6.0000.5257), and the study was registered at the Brazilian Registry of Clinical Trials (ReBEC, identifier number RBR-33dd4p4, available at https://ensaiosclinicos.gov.br ). The study started recruitment in September 2019 and ended in December 2022.

### BoNT-A treatment guided by US or EMG

The choice of injected muscles for each patient was carefully planned, taking into account several important variables. This included a physical examination that assessed the phenomenology of movement, the degree of muscle contraction measured through EMG, and the US aspects of the dystonic muscle, such as the presence of hypertrophy and visible muscle contraction at rest.

The US-guided injections were performed using the LOGIQ machine (GE Healthcare) and a 7.5-Mhz linear transducer. The muscles were scanned in the axial plane, and the injection was performed using an out-of-plane technique. The needle was inserted into the skin and accompanied by direct US muscle visualization. After the target muscle was identified, the BoNT-A units were injected safely according to each patient's programed dose. One or more injection points were performed according to the size of the muscle and with the purpose of spreading the toxin. Doppler mode could be used, if necessary, to assess vascular structures, specifically to visualize the vertebral artery in obliquus capitis inferior (OCI) muscle injections. The EMG-guided injections were performed using the MyoGuide system (Intronix Technologies Corp.). The injections were performed with a sterile monopolar needle with 0.40 mm in diameter and 25 mm in length, which works as an electrode and injection needle. Prior to needle insertion, the skin was cleaned per the institutional protocol. The needle insertion point was determined by palpation and anatomical references. Muscle activity was graded according to the intensity of the sound emitted by the device, which represents the degree of abnormal muscle contraction at rest. Through this subjective grading, the muscles were selected, and BoNT-A doses were calculated and injected. This process was repeated in each dystonic muscle and at multiple points according to each patient's individual protocol.

### Outcome assessment

The TWSTRS was applied immediately before the BoNT-A injection and after 4 to 6 weeks by a single independent examiner unaware of the randomized group and who did not participate in the BoNT-A treatment. This scale consists of a score that ranges from 0 to 85 points. It comprises an investigator-rated severity subscale (score: 0–35 points) and 2 patient-rated disability and pain subscales (score: 0–30 and 0–20 points, respectively). 15 16 17 The TWSTRS score was evaluated before and after the procedure and included the clinical evaluation of the phenomenology of movement and classification of disease severity by a neurologist expert in movement disorders and the patients' self-impression of the impact of dystonia on activities of daily living and instrumental activities of daily living, as well as disability secondary to pain. Both absolute and relative percentage changes before and after the BoNT-A treatment were calculated. The primary outcome was the difference in change between the two groups (US and EMG) in the total TWSTRS score and the scores on its separate components (severity, incapacity and pain).

### Statistical analysis

The continuous data were expressed as mean and standard deviation (SD) values or 95%CIs, and the categorical data, as proportions. The sample size calculation aimed to detect a clinically-meaningful difference of at least 6 points in the reductions in the TWSTRS scores between the treatment guided by US and EMG, 24 with an estimated SD of 6 points, an α error of 0.05, and a statistical power of 0.85. We considered clinically-significant reductions those is in the range from 6 to 10 points; most previous studies demonstrated an 8-point drop. 25 Because currently there is no evidence of superiority of one guiding procedure over the other, we did not establish an a-priori reference procedure, and designed a traditional two-tailed superiority trial, without prespecifying non-inferiority margins. If it existed, this 6-point difference between TWSTRS score changes would be detected regardless of which procedure (US or EMG) would be the best. A total of 38 randomized patients (19 in each group) would be necessary. We finished by randomizing 40 individuals, 20 in each group. Comparisons of baseline clinico-demographic characteristics of the participants in the two groups were performed by Mann-Whitney tests for continuous variables and by Fisher's exact tests for dichotomous variables. Intragroup analysis of changes in pre- and posttreatment TWSTRS scores was performed by Wilcoxon signed rank tests, and comparisons of changes between the US and EMG groups were performed by Mann-Whitney tests for continuous absolute and relative TWSTRS score changes, and by Fisher's exact tests for dichotomized changes (> 30% relative reduction and < 20 points in posttreatment TWSTRS). Changes in the separate components of the TWSTRS were also evaluated by the same statistical tests. All statistical analyses were based on the intention-to-treat principle, and they were performed using the IBM SPSS Statistics for Windows (IBM Corp.) software, version 19.0. A 2-tailed *p* -value < 0.05 was regarded as significant.

## RESULTS

A total of 40 patients (mean age: 54 years; 45% of female subjects) with idiopathic CD were randomized to receive BoNT-A injection treatment either guided by US (20 patients) or by EMG (20 patients). Table 1 outlines the main clinico-demographic characteristics of the participants. The two groups were well balanced regarding all baseline characteristics, except for the fact that the participants in the EMG group were on average 5 years younger than those in the US group, which was not statistically significant. Most importantly, the total and component TWSTRS scores were equal between the two groups, as well as the number of injections and the total dose of BoNT-A administered.

**Table 1:** Main clinico-demographic characteristics of the study sample

Characteristics		US group ( *n* = 20)	EMG group ( *n* = 20)	*p* -value
Mean age (years)		56.7 ± 13.7	51.6 ± 14.8	0.27
Female sex: n (%)		10 (50%)	8 (40%)	0.75
White race/ethnicity: n (%)		11 (55%)	13 (65%)	0.75
Mean disease duration (years)		10.7 ± 6.6	10.2 ± 5.4	0.80
Adjuvant treatment: n (%)	Analgesics	1 (5%)	1 (5%)	1.00
	Myorelaxants	4 (20%)	4 (20%)	1.00
	Anticholinergics	4 (20%)	4 (20%)	1.00
	Benzodiazepines	9 (45%)	8 (40%)	1.00
TWSTRS score	Total	35 ± 12	36 ± 15	0.95
	Severity	17 ± 8	18 ± 8	0.51
	Disability	10 ± 7	8 ± 5	0.44
	Pain	9 ± 4	9 ± 6	0.71
Mean number of BoNT-A injections		11 ± 7	10 ± 6	0.83
Mean total BoNT-A dose (UI)		417 ± 215	407 ± 150	0.98

Abbreviations: BoNT-A, botulinum toxin type A; EMG, electromyography; TWSTRS, Toronto Western Spasmodic Torticollis Rating Scale; US, ultrasound.

Note: The values presented were compared by Mann-Whitney tests or Fisher's exact tests.

In the current study, 5 patients (2 in the UG group and 3 in the EMG group) without previous treatment (“naïve”) were included. The other participants were undergoing regular treatment, with the last application being at least 16 weeks prior, and previous application methods were not considered. The sample consisted of patients who had previously undergone application guided by US, EMG, or only by anatomical landmarks. The methodology was consistent with that of most, 8 9 10 11 which included naïve patients or those undergoing treatment with BoNT-A—without application in the last 16 to 18 weeks—or did not make a distinction.

In general, the dystonias in both groups were characterized by movements such as rotation and lateralization of the neck, as well as shoulder elevation. In the US group, cases of anterocollis, lateral shift, and sagittal shift were observed. In the EMG group, the variety of dystonias was somewhat greater, with more cases of anterocollis, retrocollis, and different types of shifts.

Table 2 shows the total number of muscles treated and the specific injected muscles and the respective BoNT-A doses in both groups. The total number of injected muscles and most of the specific muscles and the respective BoNT-A doses were not different between the groups. The exceptions were the OCI and the semispinalis capitis et cervicis muscles, which were only injected in the US group.

**Table 2:** Injected muscles and botulinum toxin type A doses

Injected muscles	US group ( *n* = 20)	EMG group ( *n* = 20)	*p* -value
Mean number of injected muscles per individual	3.3 ± 1.0	3.0 ± 0.6	0.16
Levator scapulae: n (%)	15 (75%)	17 (85%)	0.70
Mean BoNT-A dose (UI)	143 ± 50	137 ± 42	0.59
Obliquus capitis inferior: n (%)	5 (25%)	0	0.047
Mean BoNT-A dose (UI)	54 ± 29	–	–
Scaleni, n (%)	0	1 (5%)	1.00
BoNT-A dose (UI)	–	50	–
Semispinalis capitis et cervicis, n (%)	8 (40%)	0	0.003
Mean BoNT-A dose (UI)	88 ± 50)	–	–
Splenius capitis et cervicis, n (%)	17 (85%)	19 (95%)	0.61
Mean BoNT-A dose (UI)	90 ± 43)	109 ± 33)	0.14
Sternocleidomastoideus, n (%)	13 (65%)	13 (65%)	1.00
Mean BoNT-A dose (UI)	79 ± 37)	115 ± 72)	0.16
Trapezius, n (%)	8 (40%)	9 (45%)	1.00
Mean BoNT-A dose (UI)	125 ± 46)	111 ± 60)	0.52

Abbreviations: BoNT-A, botulinum toxin A; EMG, electromyography; US, ultrasound.

Note: The values presented were compared by Mann-Whitney tests or by Fisher's exact tests.

Table 3 presents the pre- and posttreatment total and component TWSTRS scores in the two groups and their respective reductions. Both groups presented a significant reduction in the total and component scores after the BoNT-A treatment. The mean absolute reduction in the total score was of 8 (95%CI: 4–12) points, which corresponded to a relative reduction of 22.5% (95%CI: 15.8–29.2%). The absolute and relative reductions in the total score were not different in the two groups (mean absolute difference of 0.1 point greater in the US group; 95%CI: −5.6– + 5.5 points; *p* = 0.97; which corresponded to a mean relative difference of 2.0%; 95% CI: −11.9– + 15.9%; *p* = 0.87). When the improvements in the total score were dichotomized as a > 30% reduction and as a posttreatment score < 20 points, neither were there differences between the groups. The same equivalence between US- and EMG-guided BoNT-A administration was also observed in the reductions in the component scores.

**Table 3:** Total and component TWSTRS scores before and after treatment

	US group ( *n* = 20)				EMG group ( *n* = 20)				Mean difference in reduction (95%CI)	*p* -value
	Treatment		Mean reduction (95%CI)	*p* -value	Treatment		Mean reduction (95%CI)	*p* -value		
	Pre	Post			Pre	Post				
Mean total TWSTRS score	35 ± 12	27 ± 13	8.3 (4.2–12.4)	< 0.001	36 ± 15	2712	8.2 (4.2–12.3)	< 0.001	0.1 (−5.6–5.5)	0.97
Relative TWSTRS reduction (%)	–	–	23.5 (13.1–33.9)	–	–	–	21.5 (13.1–29.9)	–	2.0 (−11.9–15.9)	0.89
Reduction in TWSTRS > 30%: n (%)	–	–	6 (30%)	–	–	–	6 (30%)	–	–	1.00
Posttreatment TWSTRS < 20: n (%)	–	–	5 (25%)	–	–	–	4 (20%)	–	–	1.00
Mean TWSTRS score: severity	17 ± 8	15 ± 6	2.0 (0.3–3.7)	0.002	18 ± 8	16 ± 8	2.6 (1.4–3.9)	0.001	0.6 (−1.4–2.7)	0.60
Mean TWSTRS score: disability	10 ± 7	7 ± 5	3.6 (1.5–5.8)	< 0.001	8 ± 5	6 ± 4	2.6 (1.2–3.9)	0.002	1.0 (−1.3–3.5)	0.37
Mean TWSTRS score: pain	9 ± 4	6 ± 4	2.5 (0.9–4.2)	0.004	9 ± 6	6 ± 5	3.1 (1.0–5.2)	0.003	0.6 (−2.1–3.2)	0.67

Abbreviations: EMG, electromyography; TWSTRS, Toronto Western Spasmodic Torticollis Rating Scale; US, ultrasound.

Notes: Intragroup comparisons were performed through Wilcoxon signed rank tests, and intergroup comparisons, through Mann-Whitney tests and Fisher's exact tests.

## DISCUSSION

To the best of our knowledge, the current is the first head-to-head randomized trial comparing two BoNT-A guiding techniques in patients with idiopathic CD. Previous studies 8 9 10 11 12 13 14 17 19 26 27 28 29 have shown greater effectiveness in the application of BoNT-A guided by some complementary method compared with anatomical guidance. We demonstrated that there was no evidence of any difference in improvements 4 to 6 weeks after the BoNT-A treatment, as assessed by the reductions in the TWSTRS scores in both groups.

Very few previous studies compared two methods of guiding BoNT injections. In 2009, Lee et al. 30 reported their initial experience with US and computed tomography (CT) as guides for BoNT-A injections in patients with CD. In their study, 30 8 patients with idiopathic CD were evaluated by a physician with 15 years of experience in movement disorders through a physical examination to classify the dystonic pattern, EMG, and PET-CT; 6 patients underwent US-guided injections, and 3 patients, CT-guided injections. The clinical outcome was assessed using the scores on the Tsui Scale and on the TWSTRS, which were evaluated 4 weeks after the last BoNT-A injection, regardless of the method used. All 8 patients experienced a marked reduction in pain and improved neck movement. Based on the TWSTRS subscale scores, severity, disability, and pain, the reduction rates were of 0.14, 0.21, and 0.16 respectively. 30 A recent, single-blinded, randomized study 31 evaluated 19 participants, 10 with spasticity of the upper limbs and 9 with focal hand dystonia, comparing BoNT injection guided by EMG using electrical stimulation (E-stim) and US. The primary outcome was the improvement in the severity of dystonia or spasticity on the visual analog scale (VAS, score range: 0–100) measured 1 month after each injection. The secondary outcome was participant discomfort, also assessed through the VAS. The benefit was equivalent between the two techniques (VAS least squares mean [LSmean]: 51.5 mm with US and 53.1 with E-stim). The E-stim was perceived as more uncomfortable by participants (VAS LSmean: 34.5 versus 19.9 for E-stim and US respectively). 31 In a randomized controlled trial, 8 68 patients with CD received BoNT-A guided by palpation or EMG. The primary endpoint was defined as the difference in the Tsui score between groups at 16 weeks. The CD patients treated with EMG guidance experienced a prolonged benefit as measured by the Tsui scale when compared with CD patients treated with palpation guidance alone. 8

The success of BoNT procedures depends on many factors, including: recognition of the clinical pattern, identification of active muscles, dose of BoNT, location, and safe injection of the target muscles. In CD patients, the treatment of anterocollis (anterior flexion of the neck) and anterocaput (frontal flexion of the head) is undeniably challenging. The longus colli (LoCol) and longus capitis (LoCap), two muscles of the deep cervical spine and flexors of the head, often contribute to these movement patterns. Ultrasound guidance provides direct visualization of the LoCol, LoCap, and other cervical muscles, as well as of adjacent structures, reducing the risks of side effects and improving the clinical response to BoNT in these conditions. The addition of EMG provides confirmation of muscular activity within the target muscle. 32

Walter et al. 28 investigated five patients with tremulous torticollis who had only a partial response to the conventional injection technique without injection of the OCI. Compared with BT injection in the superficial neck muscles, the additional US-guided injection in the OCI led to greater benefit (self-assessment of improvement in cervical dystonia; *p* = 0.026; Mann-Whitney test), especially for the tremulous component ( *p* = 0.007), although the total dose of BoNT was not altered. 28

In the current study, the OCI and the semispinalis capitis and cervicis muscles were only treated in the US group ( Table 4 ). These muscles are located in deeper planes, and their identification is only possible through an imaging method. Walter et al. 28 demonstrated that the additional injection in the OCI muscle, when compared with the injection only in the superficial muscles, yielded greater benefits, mainly in the fluttering component of CD. However, despite this potential advantage of the US- over the EMG-guiding technique in the administration of BoNT-A to these muscles, our results did not demonstrate any statistical or clinically significant difference between the two groups.

**Table 4:** Injected muscles in the electromyography and ultrasound groups

	Number of participants	
Muscles	Ultrasound	Electromyography
Levator scapulae	15	17
Obliquus capitis inferior	5	0
Scaleni	0	1
Semispinalis capitis and cervicis	8	0
Splenius capitis and cervicis	17	18
Sternocleidomastoideus	13	13
Trapezius	8	9

A study 18 compared the prevalence of dysphagia after the application of BoNT guided by isolated EMG and by EMG associated with US in 5 CD patients. It demonstrated a prevalence of 34.7% of dysphagia with the application through isolated EMG and no episodes of dysphagia when US was associated with EMG. 18 In the present study, no complications such as dysphagia, excessive muscle weakness, or inadvertent injury to noble structures were observed, corroborating the hypothesis of the previously-cited study, 18 that using an imaging method reduces the risk of complications inherent to both the injection and the toxin.

The current study has some limitations that warrant discussion. First, it was a single-center trial with a relatively small sample of CD patients. With our sample, we were able to demonstrate differences, if they existed, of at least 6 points in improvements on the TWSTRS score between US- and EMG-guided BoNT-A treatments. We found a ≤ 1 point difference between the two groups, suggesting that our results are consistent in supporting the lack of evidence of any difference in improvements between the two methods. However, the present study shall be considered a pilot, hypothesis-generating, clinical trial, and our findings shall be confirmed in larger, possibly multicenter, trials. Second, both methods herein assessed are highly operator-dependent. In the current study, all injections were performed by the same independent neurologist, experienced in both methods. However, these results may vary according to different examiners. Finally, our patients were mainly middle-aged with a mean disease duration of 11 years; hence, our findings may not be generalizable to younger patients or to those with a shorter duration of CD. In addition, the patients evaluated were undergoing regular BoNT-A treatment, they were not treatment naïve. The results herein obtained were intended to demonstrate the superiority of one of the application methods over the other, not the response to treatment with BTxA, which is already well-established.

In conclusion, the present randomized clinical trial provided no evidence of any significant differences in improvements, assessed by the validated TWSTRS between US- and EMG-guided techniques for the BoNT-A treatment of CD.
